# Immune characteristics of kidney transplant recipients with acute respiratory distress syndrome induced by COVID-19 at single-cell resolution

**DOI:** 10.1186/s12931-024-02682-9

**Published:** 2024-01-18

**Authors:** Junyu Lu, Yin Chen, Kaihuan Zhou, Yicong Ling, Qianqian Qin, Weisheng Lu, Lian Qin, Chenglin Mou, Jianfeng Zhang, Xiaowen Zheng, Ke Qin

**Affiliations:** 1 Present Address: Intensive Care Unit, The Second Affiliated Hospital of Guangxi Medical University, Nanning, 530007 China; 2 Present Address: Department of Emergency Medicine, The Second Affiliated Hospital of Guangxi Medical University, Nanning, 530007 China; 3 Guangxi Health Commission Key Laboratory of Emergency and Critical Medicine, Nanning, 530007 China; 4 Department of Anesthesiology, The Second Affiliated Hospital of Guangxi Medical University, Nanning, 530007 Guangxi China; 5 Department of Anesthesiology, Guilin People’s Hospital, Guilin, 541002 China

**Keywords:** COVID-19, Acute respiratory distress syndrome, Kidney transplant recipient, scRNA-seq, Immunosuppression, Innate immunity

## Abstract

**Background:**

COVID-19-induced acute respiratory distress syndrome (ARDS) can result in tissue damage and multiple organ dysfunction, especially in kidney transplant recipients (KTRs) receiving immunosuppressive drugs. Presently, single-cell research on COVID-19-induced ARDS is considerably advanced, yet knowledge about ARDS in KTRs is still constrained.

**Methods:**

Single-cell RNA sequencing (scRNA-seq) analysis was performed to construct a comprehensive single-cell immune landscape of the peripheral blood mononuclear cells (PBMCs) of eight patients with COVID-19-induced ARDS, five KTRs with COVID-19-induced ARDS, and five healthy individuals. Subsequently, we conducted a comprehensive bioinformatics analysis, including cell clustering, enrichment analysis, trajectory analysis, gene regulatory network analysis, and cell–cell interaction analysis, to investigate the heterogeneity of the immune microenvironment in KTRs with ARDS.

**Result:**

Our study revealed that KTRs exhibit significant heterogeneity with COVID-19-induced ARDS compared with those of other individuals, with significant reductions in T cells, as well as an abnormal proliferation of B cells and monocytes. In the context of dual influences from immunosuppression and viral infection, KTRs exhibited more specific plasma cells, along with significant enrichment of dysfunctional GZMB and XAF1 double-positive effector T cells and IFI27-positive monocytes. Additionally, robust communication existed among T cells and monocytes in cytokine signaling. These effects impede the process of immune reconstitution in KTR patients.

**Conclusion:**

Our findings suggest that KTRs with COVID-19-induced ARDS show elevated antibody levels, impaired T cell differentiation, and dysregulation of innate immunity. In summary, this study provides a theoretical foundation for a comprehensive understanding of COVID-19-induced ARDS in KTRs.

**Supplementary Information:**

The online version contains supplementary material available at 10.1186/s12931-024-02682-9.

## Introduction

COVID-19 is primarily transmitted through droplets or direct contact. It infects the respiratory tract, causing pneumonia in most cases and acute respiratory distress syndrome (ARDS) in around 15% of cases [44]. In the early disease stages in adults, there is a notable reduction in the number of CD8^+^ and CD4^+^ T cells [42]. Decreases in these cells are accompanied by accelerated viral replication, which triggers chemotactic reactions and inflammatory infiltration [42]. ARDS typically develops within 7–10 days following disease onset [42], and is the underlying pathology of severe COVID-19, primarily resulting from elevated levels of pro-inflammatory cytokines; this is commonly known as a cytokine storm [39, 63]. The severity and mortality of COVID-19 are mainly due to sepsis, multiple organ failure caused by cytokine storms, and respiratory failure caused by acute lung injury and ARDS [21, 24, 43, 46, 63]. In a study of 204 COVID-19 patients who received systemic glucocorticoids, the mortality rate was low, with only 5 deaths recorded [21]. However, the mortality rate among COVID-19 patients who develop ARDS is usually closer to 70% [68]. Therefore, it is crucial to investigate the mechanisms of immune dysregulation in COVID-19-induced ARDS to improve the management and prognosis.

Given the potential risks of infection, complications, and immunosuppressive exposure faced by kidney transplant recipients (KTRs) [3], SARS-CoV-2 infection significantly increases patient mortality risk [46]. COVID-19 has a higher likelihood of inducing moderate to severe pneumonia in individuals who have received kidney transplants [1, 12]. Despite the significant therapeutic effects of COVID-19 vaccines in the general population, vaccinated KTRs still face the risk of breakthrough infection, with a mortality risk 8.1 times higher than that in the general population [5, 11, 23]. Hence, exploring the single-cell immune profile of KTRs with COVID-19-induced ARDS, particularly in regards to the impact of virus infection and immunosuppression, is crucial for effectively managing the disease and promoting recovery in patients.

In this study, we constructed a single-cell atlas of immune cells in the peripheral blood mononuclear cells (PBMCs) of both KTRs and non-KTRs with COVID-19-induced ARDS. Additionally, we analyzed the dynamic changes in specific subpopulations of different immune cells, as well as changes in the implicated signaling pathways, revealing the developmental trajectories of different cell subpopulations and their primary regulatory targets at the transcriptional level.

## Methods

### Sample groups

This study adhered to the principles outlined in the Declaration of Helsinki. The participants provided written informed consent prior to sample collection, following standard procedures. Eighteen peripheral blood samples were collected and analyzed in this study. The sample groups consisted of eight patients with COVID-19-induced ARDS who had not received a kidney transplant, five KTRs with COVID-19-induced ARDS, and five healthy individuals (Additional file 2: Table S1).

### PBMC collection and processing

Human peripheral blood (20 mL) was collected in ethylenediaminetetraacetic acid anticoagulation tubes and centrifuged at 2000 rpm for 10 min at room temperature (27 °C) to separate the plasma. Lymphocyte separation solution (5 mL) [62] was added to each tube, samples were centrifuged, and the upper layer of plasma was aspirated [28]. An equal volume of sterile phosphate-buffered saline (PBS) was added to dilute the remaining blood cells [22]. The diluted blood cells were combined with the upper layer of the lymphocyte separation solution in a 1:1 ratio and centrifuged at 2500 rpm for 20 min. The resultant middle white film layer was collected into a new 10 mL centrifuge tube and PBS was added to a total volume of 10 mL. The mixture was then centrifuged at 2000 rpm at room temperature for 5 min, after which the lymphocyte separation solution was removed. PBS (1 mL) was added to dilute the resuspended cells to a fixed volume of 10 mL, followed by centrifugation at 1,200 rpm at room temperature for 5 min. The supernatant was discarded, and the separated PBMC precipitate was resuspended in a cell lyophilization solution.

## Single-cell RNA sequencing

The DNBelab C Series Single-Cell Library Prep Set (MGI Tech, Shenzhen, China) was used to prepare single-cell RNA-seq (scRNA-seq) libraries, following an established method. Briefly, single-cell suspensions were subjected to droplet encapsulation, emulsion breakage, collection of mRNA-captured beads, reverse transcription, cDNA amplification, and purification, resulting in the generation of barcoded scRNA-seq libraries. Indexed sequencing libraries were constructed according to the manufacturer’s instructions. Sequencing libraries were quantified using the Qubit ssDNA Assay Kit (Thermo Fisher Scientific, Waltham, MA, USA). The read structure was paired-end: Read 1 covered 30 bases and encompassed two 10-bp cell barcodes and a 10-bp unique molecular identifier; Read 2 comprised 100 bases of transcript sequences along with a 10-bp sample index. Quality control was performed on the sequencing data to filter out cells that were in the highest and lowest 1% of gene expression, as well as cells with mitochondrial gene expression exceeding 10%.

### ScRNA-seq clustering

The Seurat package [9] was used to perform various analyses, including data integration, cell clustering, identification of clustering results, and generation of comprehensive single-cell maps. The dimensionality of the results was reduced using a consistent manifold approximation and projection (UMAP) algorithm [6]. Cell clusters were manually annotated using cell type information from cell marker databases, laboratory experiments, and previous studies [25, 45]. The Seurat package [9] was also used to identify subpopulations in the clustering analysis. The subpopulations were manually annotated by incorporating gene markers associated with the disease to obtain detailed subpopulation information.

### Enrichment, trajectory, gene regulatory network, and intercellular communication analyses

The clusterProfiler package [64] was used to conduct enrichment analysis of subpopulation genes with respect to Gene Ontology and Kyoto Encyclopedia of Genes and Genomes to reveal the biological significance of these subpopulations in relation to cellular functions and disease development. The R package Monocle3 [53] was used to construct developmental trajectories for individual cell subpopulations to investigate cell development, differentiation, and transcriptional dynamics. The gene regulatory network (GRN) was analyzed using the pySCENIC module with a particular emphasis on transcription factors [4], Van [57] to investigate transcription regulation, which is a crucial aspect of gene regulation involving transcription factors and their respective binding sites. The iTALK package (https://doi.org/10.1101/507871) was used to identify high-confidence ligand-receptor interactions between subpopulations of cells, thereby uncovering significant events in intercellular communication.

### Statistical analysis

Statistical analysis was performed on the bioinformatics cloud platform BioinforCloud (http://www.bioinforcloud.org.cn). Statistical significance was set at *P-*valve < 0.05.

## Results

### Immune cells in PBMCs of COVID-19-induced ARDS patients

**Fig. 1:**
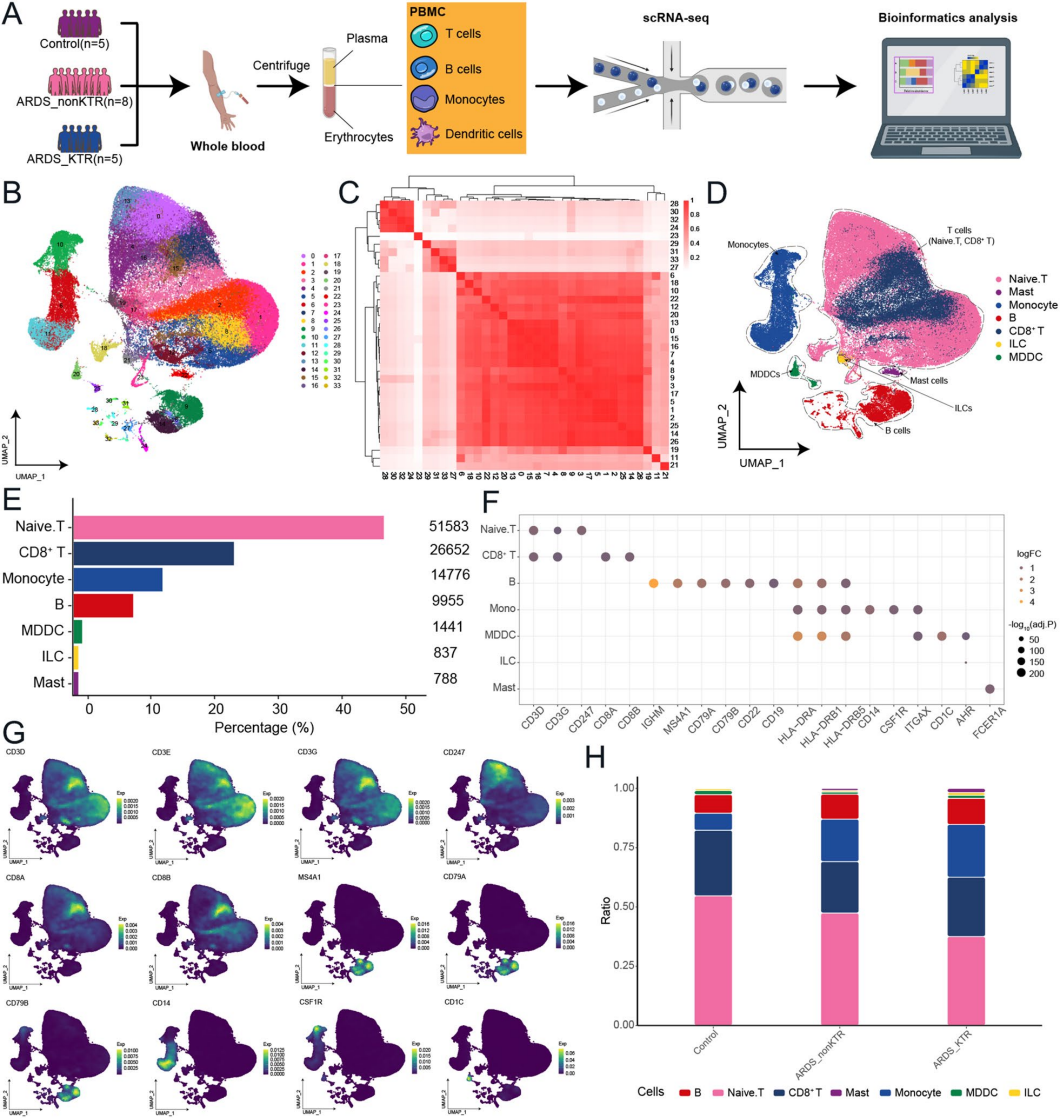
Single-cell transcriptional analysis of PBMCs in ARDS patients induced by COVID-19. **A** Workflow of overall study. **B** Single-cell atlas of ARDS induced by COVID-19, with a total of 33 cell clusters of 106,033 cells captured. **C** The correlations among different clusters of single cells. **D**, **E** Identification of the 33 clusters with various cell types, including naïve T cells (n = 51,583 cells), B cells (n = 9,955 cells), CD8^+^ T cells (n = 26,652 cells), monocytes (n = 14,776 cells), monocyte-derived dendritic cells (MDDCs; n = 1,441 cells), innate lymphoid cells (ILCs; n = 837 cells), and mast cells (n = 788 cells). **F** Bubble chart illustrating the expression of marker genes for each individual cell type. The colors of the bubbles indicate the logFC and the sizes of the bubbles represent -log10(adj.P). **G** Cell clusters with marker genes mapped in the single-cell spectrum for naïve T cells (CD3D, CD3E, CD3G, and CD247), CD8^+^ T cells (CD8A and CD8B), B cells (MS4A1, CD79A, and CD79B), monocytes (CD14 and CSF1R), and MDDCs (CD1C). **H** Cellular components and changes in the control group (Control), non-kidney transplant recipients (KTRs) with ARDS group (ARDS_nonKTR), and KTRs with ARDS group (ARDS_KTRs)

Sequencing analysis was performed on eighteen individuals, including eight patients with COVID-19-induced ARDS (ARDS_nonKTR; n = 36,466 single cells), five KTRs with COVID-19-induced ARDS (ARDS_KTR; n = 23,980), and five healthy controls (Control; n = 50,752) (Fig. 1A). After quality control and preprocessing, we identified 106,033 high-quality single-cell transcriptomes. These transcriptomes were categorized into 33 clusters and visualized on a single-cell map (Fig. 1B, C). We examined the correlations between the expression patterns among the clusters and their respective cell markers, and seven distinct cell types were identified: CD8^+^ T cells, naïve T cells, B cells, innate lymphoid cells (ILCs), monocytes, monocyte-derived dendritic cells (MDDCs), and mast cells (Fig. 1D–G). Compared to the control group, ARDS patients had a higher abundance of B cells, monocytes, and mast cells and a lower abundance of naïve T cells and CD8^+^ T cells (Fig. 1H), consistent with a previous study [35]. Compared with the ARDS_nonKTR group, the ARDS_KTR group showed a higher abundance of B cells, CD8^+^ T cells, monocytes, MDDCs, ILCs, and mast cells.

### Abnormal proliferation of plasma cells in KTRs with ARDS

**Fig. 2:**
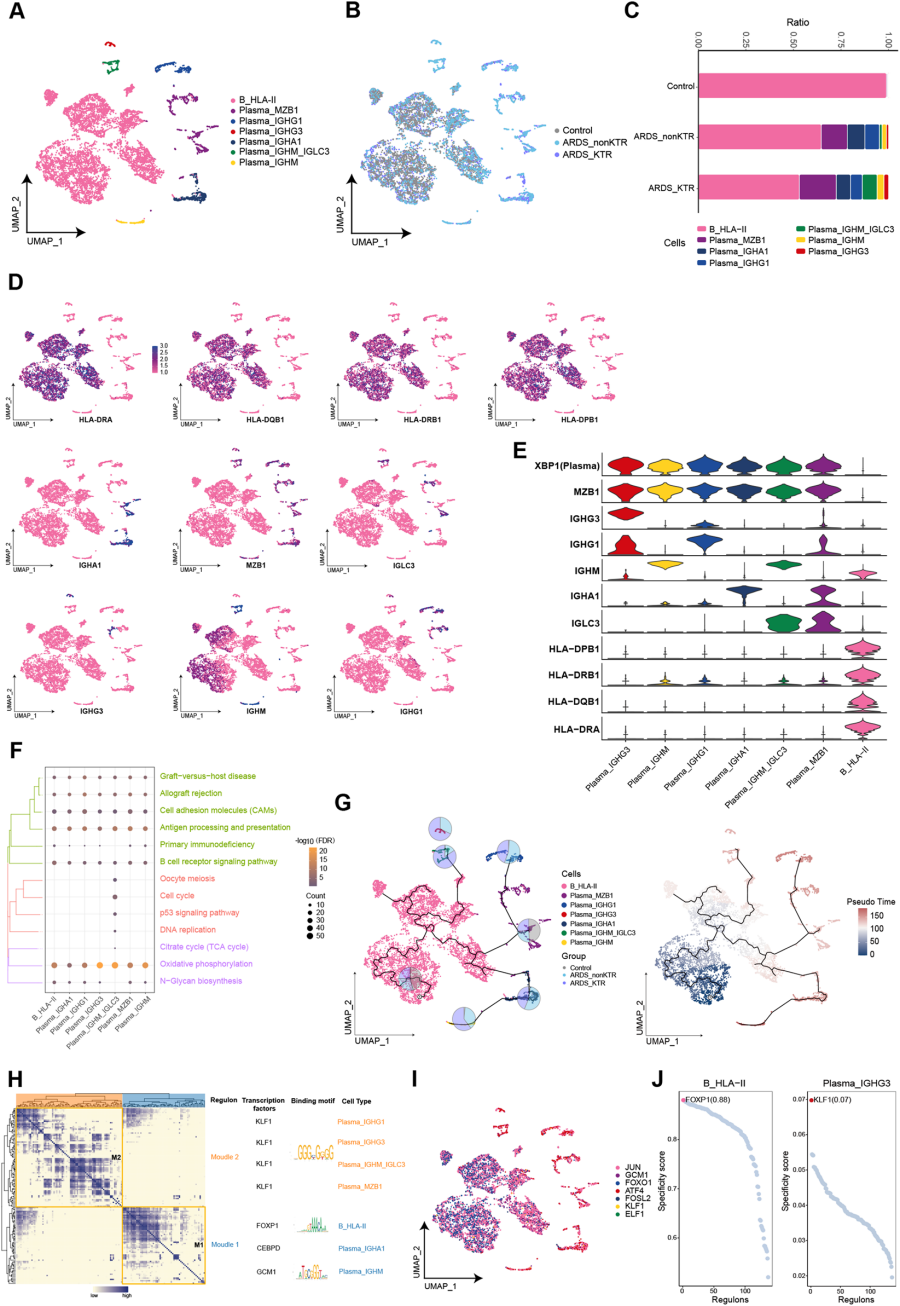
Heterogeneity of B cell subpopulations among ARDS patients and KTRs with ARDS. **A**, **B** Composition and single-cell maps of B cells in patients with ARDS. **C** The dynamic changes of B cell subpopulations in patients with ARDS. **D**, **E** Expression of marker genes in B cell subpopulations. **F** Bubble chart illustrating the signaling pathways associated with B cell subpopulations. **G** Single-cell trajectory analysis of B cell subpopulations. **H** Motif module heatmap representing the B cell subpopulations. **I** Transcription factors present in B cell subpopulations. **J** Transcription factor activities in B_HLA-II and Plasma_IGHG3

We depicted a single-cell atlas of B cells that identified eight subpopulations with varied abundance among patients (Fig. 2A, B). By examining the changes in abundance in B cell subpopulations, we found that B_HLA-II (HLA-DRA, HLA-DQB1, HLA-DRB1, and HLA-DPB1) subpopulations were the primary B cell components of the control group (Fig. 2C–E). Several plasma cell subpopulations were prominently enriched in patients compared with the control group, especially those in the ARDS_KTR group. In comparison to the ARDS_nonKTR group, the IGHG3 high-expression subpopulation Plasma_IGHG3 exhibited a higher abundance in the ARDS_KTR group. Enrichment analysis indicated that multiple B cell subpopulations were significantly enriched in oxidative phosphorylation, antigen processing and presentation, and the B cell receptor signaling pathway (Fig. 2F). In the differentiation trajectory of the B cell subpopulations, we observed that the B_HLA-II subpopulation resided at the commencement of B cell development and gave rise to distinct subpopulations (Fig. 2G). GRN analysis revealed that these cellular subpopulations were segregated into two modules and governed by the transcription factors KLF1, FOXP1, CEBPD, and GCM1 (Fig. 2H–J).

### PCNA-positive naïve T cell subpopulation was significantly enriched in KTRs with ARDS

**Fig. 3:**
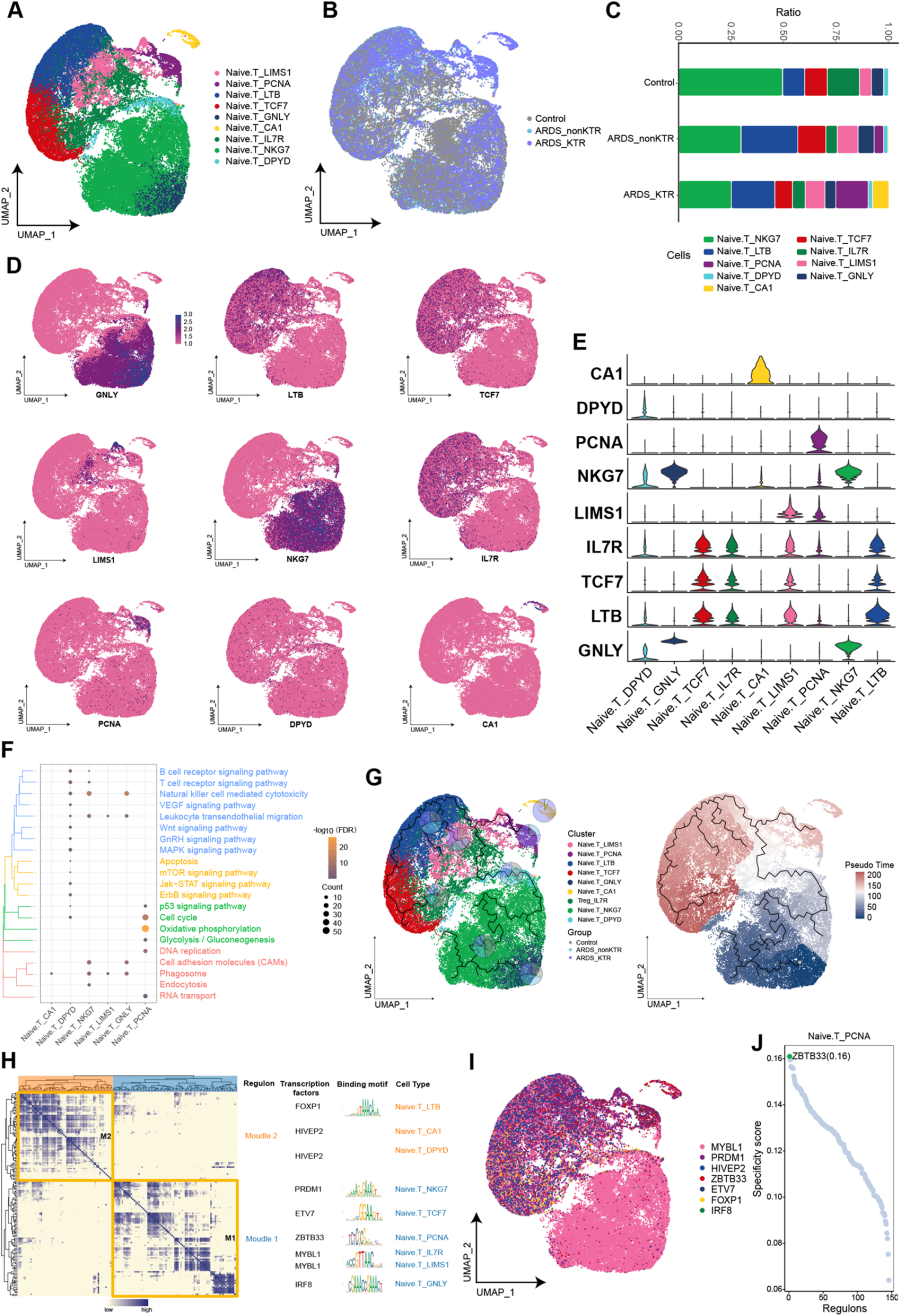
Heterogeneity of naïve T cell subpopulations among ARDS patients and KTRs with ARDS. **A**, **B** Composition and single-cell maps of naïve T cells in patients with ARDS. **C** The dynamic changes of naïve T cell subpopulations in patients with ARDS. **D**, **E** Expression of marker genes in naïve T cell subpopulations. **F** Bubble chart illustrating the signaling pathways associated with naïve T cell subpopulations. (**G**) Single-cell trajectory analysis of naïve T cell subpopulations. **H** Motif module heatmap representing the naïve T cell subpopulations. **I** Transcription factors present in naïve T cell subpopulations. **J** Transcription factor activities in Naive.T_PCNA

We identified eight subpopulations of naïve T cells and aligned them with single-cell maps from the various patients (Fig. 3A, B). It was noteworthy that the naïve T cell subpopulation specifically expressing PCNA (Naive.T_PCNA) exhibited a distinctive enrichment in both ARDS groups (Fig. 3C–E). In the ARDS_KTR group, the Naive.T_PCNA subpopulation showed more enrichment compared with the ARDS_nonKTR group. Enrichment analysis demonstrated a significant enrichment of Naive.T_PCNA in pathways associated with oxidative phosphorylation, cell cycle, the p53 signaling pathway, and DNA replication (Fig. 3F). This observation implies that this subpopulation may play an important role in cellular energy metabolism and the proliferation processes. Using trajectory analysis, we elucidated the developmental trajectory of the naïve T cell subpopulation (Fig. 3G). Our findings revealed that Naive.T_PCNA was located at the final point of this trajectory, potentially playing a significant role in functional T cell differentiation. GRN analysis revealed that the clustering of markers in naïve T cell subpopulations could be categorized into two distinct modules (Fig. 3H). These modules were regulated by the transcription factors MYBL1, PRDM1, HIVEP2, and ZBTB33 (Fig. 3I–J).

### GZMB and XAF1 double-positive effector T cells constituted the major component of CD8^+^ T cells in KTRs with ARDS

**Fig. 4:**
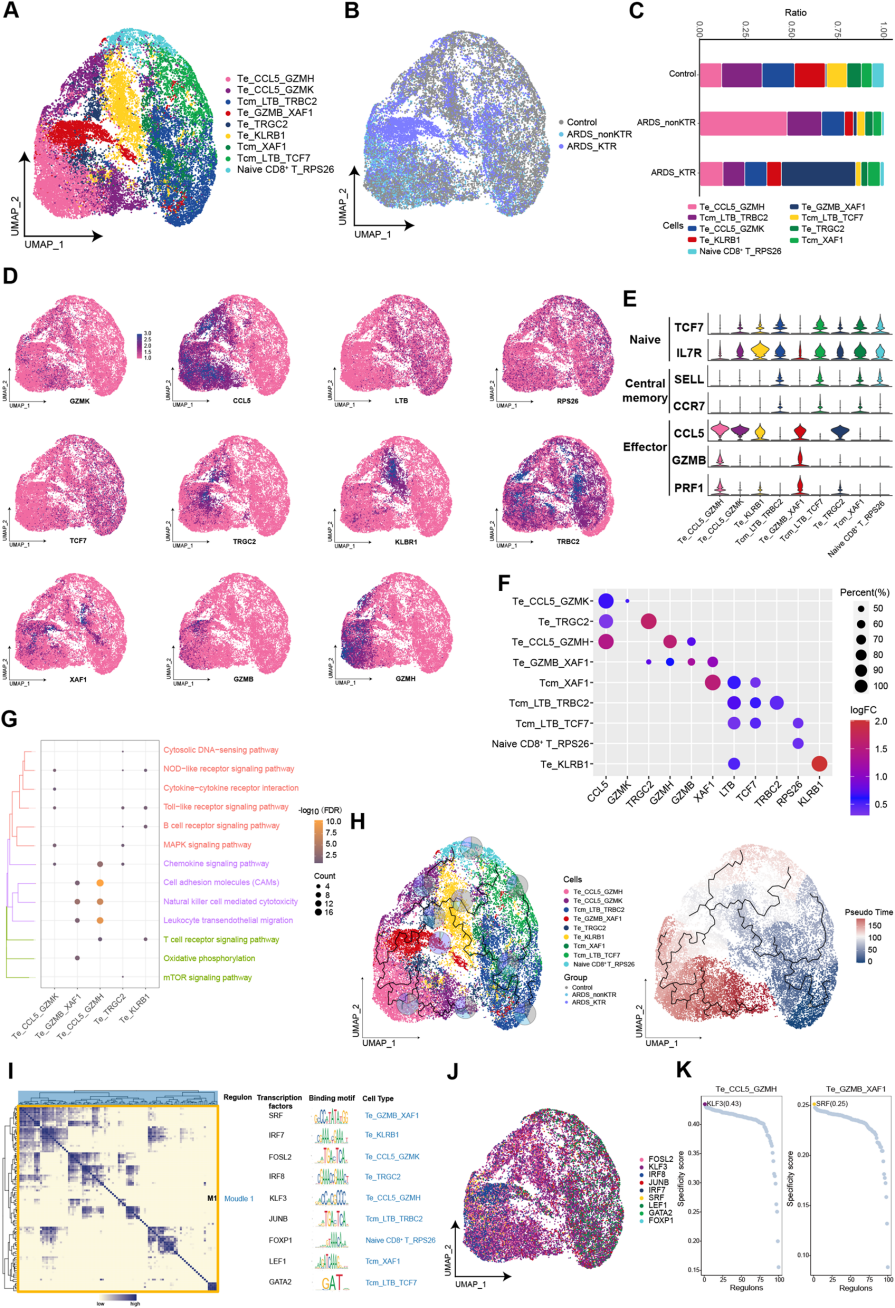
Heterogeneity of CD8^+^ T cell subpopulations among ARDS patients and KTRs with ARDS. **A**, **B** Composition and single-cell maps of CD8^+^ T cells in patients with ARDS. **C** The dynamic changes of CD8^+^ T cell subpopulations in patients with ARDS. **D** Single-cell maps reveal marker gene expression in CD8^+^ T cell subpopulations. **E** Identification of CD8^+^ T cell subpopulations based on the expression of specific biomarker genes associated with each cell subpopulation. **F** Expression of marker genes in CD8^+^ T cell subpopulations. **G** Bubble chart illustrating the signaling pathways associated with CD8^+^ T cell subpopulations. **H** Single-cell trajectory analysis of CD8^+^ T cell subpopulations. **I** Motif module heatmap representing the CD8^+^ T cell subpopulations. **J** Transcription factors present in CD8^+^ T cell subpopulations. **K** Transcription factor activities in Te_CCL5_GZMH and Te_GZMB_XAF1

A previous study has indicated that immune injury mediated by T cells could serve as a significant factor in the progression of ARDS [38]. In CD8^+^ T cells, nine cell subpopulations were identified, consisting of four effector T (Te) cell subpopulations, three central memory T (Tcm) cell subpopulations, and one naive CD8^+^ T cell subpopulations (Fig. 4A–F). Compared with the control group, there was a substantial increase in Te cells in both ARDS groups. Notably, there were differences between the ARDS_nonKTR and ARDS_KTR groups regarding the types of Te cells, which might be attributed to the immunosuppressive state of KTRs. Specifically, Te_CCL5_GZMH served as the primary component of CD8^+^ T cells in the ARDS_nonKTR group, whereas the Te_GZMB_XAF1 subpopulation was the most common in the ARDS_KTR group. These subpopulations were significantly enriched in natural killer cell-mediated cytotoxicity, leukocyte transendothelial migration, and cell adhesion molecules (Fig. 4F). Analysis of the CD8^+^ T cell developmental trajectory revealed that Te_GZMB_XAF1 was located in an intermediate state and subsequently differentiated into Te_CCL5_GZMH (Fig. 4G). This further suggests that Te_GZMB_XAF1 may be a functionally immature Te cell subpopulation. Through GRN analysis of the CD8^+^ T cell subpopulations, a module was identified that was regulated by many transcription factors, including FOSL2, SRF, JUNB, and KLF3 (Fig. 4H-J).

### Monocytes with IFI27 expression were significantly increased in KTRs with ARDS

**Fig. 5:**
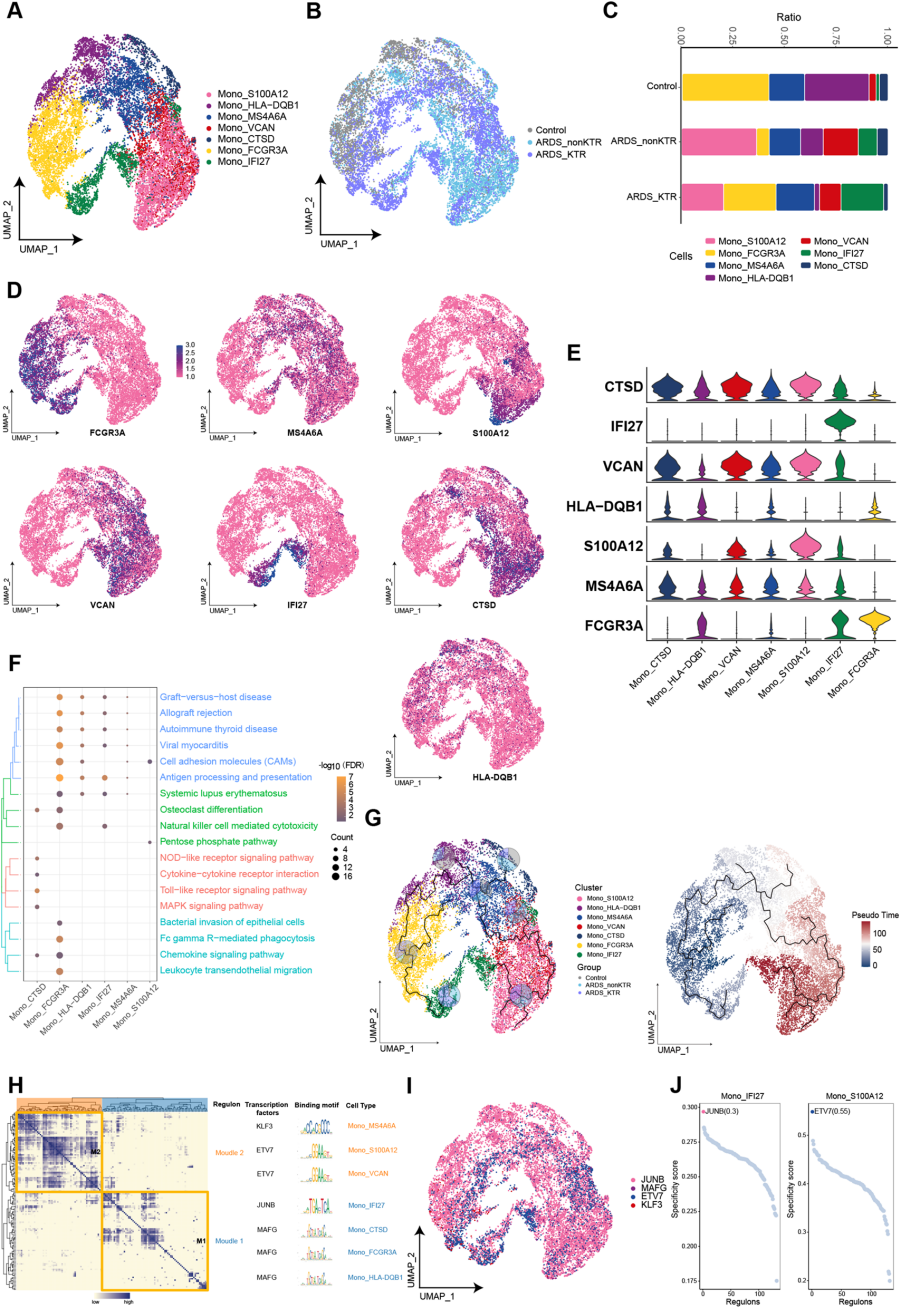
Heterogeneity of monocyte subpopulations among ARDS patients and KTRs with ARDS. **A**, **B** Composition and single-cell maps of monocytes in patients with ARDS. **C** The dynamic changes of monocyte subpopulations in patients with ARDS. **D**, **E** Expression of marker genes in monocyte subpopulations. **F** Bubble chart illustrating the signaling pathways associated with monocyte subpopulations. **G** Single-cell trajectory analysis of monocyte subpopulations. **H** Motif module heatmap representing the monocyte subpopulations. (**I**) Transcription factors present in monocyte subpopulations. **J** Transcription factor activities in Mono_IFI27 and Mono_S100A12

We identified seven monocyte subpopulations and mapped them onto the single-cell atlas (Fig. 5A, B). In contrast to the control group, Mono_S100A12 exhibited specific enrichment in both ARDS groups, particularly dominating in the ARDS_nonKTR group (Fig. 5C–E). Compared with the control and ARDS_nonKTR groups, Mono_IFI27 specifically expressing IFI27 were significantly increased in the ARDS_KTR group. The enrichment analysis showed that Mono_S100A12 was enriched in cell adhesion molecules, glycolysis/gluconeogenesis, and the Toll-like receptor signaling pathway (Fig. 5F). Within the developmental trajectory of Mono subpopulations, Mono_IFI27 was positioned at the endpoint, and was likely derived from the differentiation of Mono_S100A12 (Fig. 5G). GRN analysis revealed that the markers of the monocyte subpopulations were clustered into two modules, and mainly regulated by JUNB, MAFG, ETV7, and KLF3 (Fig. 5H–J).

### ANXA1-positive MDDCs constituted the major component of MDDCs in KTRs with ARDS

**Fig. 6:**
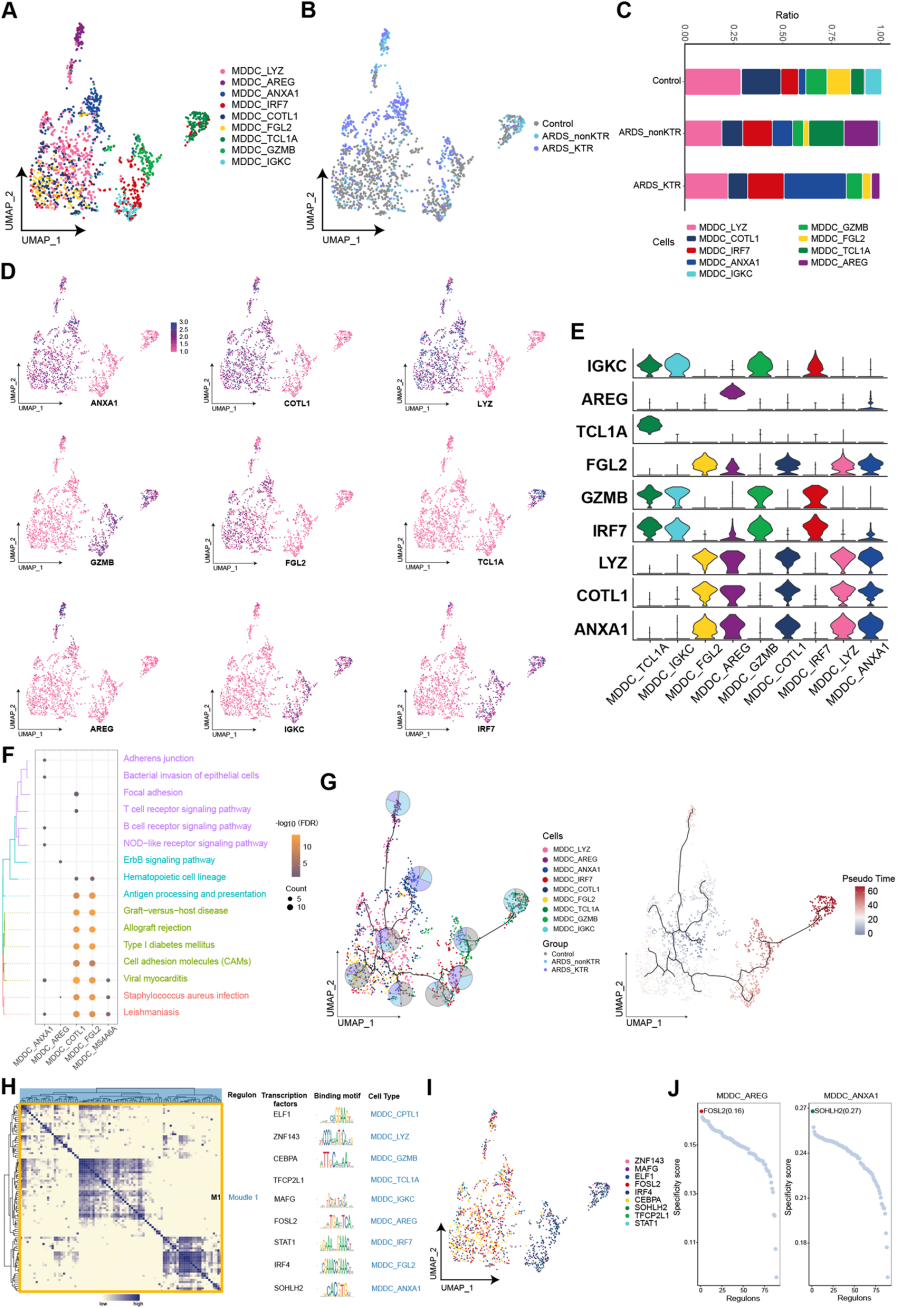
Heterogeneity of MDDC subpopulations among ARDS patients and KTRs with ARDS. **A**, **B** Composition and single-cell maps of MDDCs in patients with ARDS. **C** The dynamic changes of MDDC subpopulations in patients with ARDS. **D**, **E** Expression of marker genes in MDDC subpopulations. **F** Bubble chart illustrating the signaling pathways associated with MDDC subpopulations. **G** Single-cell trajectory analysis of MDDC subpopulations. **H** Motif module heatmap representing the MDDC subpopulations. **I** Transcription factors present in MDDC subpopulations. **J** Transcription factor activities in MDDC_AREG and MDDC_ANXA1

Nine clusters were identified from MDDCs, and the abundance of patient-specific MDDCs was estimated using UMAP (Fig. 6A, B). Compared with the control group, the MDDC_ANXA1 abundance was significantly upregulated and MDDC_AREG exhibited specific enrichment in both ARDS groups (Fig. 6C–E). Notably, MDDC_ANXA1 was the predominant MDDC component in the ARDS_KTR group. The enrichment analysis showed a significant enrichment of MDDC_ANXA1 in the adherens junction and NOD-like receptor signaling pathways, whereas MDDC_AREG was enriched in the ErbB signaling pathway (Fig. 6F). Developmental trajectory analysis revealed that both MDDC_ANXA1 and MDDC_AREG were positioned at the terminal point of the MDDC subpopulation differentiation trajectory (Fig. 6G). GRN analysis showed that markers associated with MDDC subpopulations clustered into a module, wherein ZNF143 and FOSL2 primarily regulated MDDC_ANXA1 and MDDC_AREG, respectively (Fig. 6H–J).

### Strong cytokine communication between different immune cells

To further investigate intercellular interactions among ARDS patient groups, we analyzed receptor-ligand interactions of cell cytokines (Additional file 1: Figure S1A-C). The results indicated that CCL5 was the predominant ligand for T cells during cytokine communication. In the ARDS_KTR group, T cells had strong communication with multiple monocyte subpopulations through the CCL5-CCR1 axis.

## Discussion

The SARS-CoV-2 variant triggers an immune response that can cause excessive and uncontrolled release of pro-inflammatory markers. This can lead to a cytokine storm, which is associated with the development of ARDS [13, 16, 20]. Furthermore, the pathogenesis of the virus may give rise to a distinct form of ARDS, differing from “typical” ARDS [48], particularly in KTRs with COVID-19-induced ARDS.

These cytokine storms may lead to the proliferation and activation of monocytes and B cells in blood of ARDS patients. Furthermore, T cells decrease significantly in patients, indicating potential functional impairment and limited T cell proliferation under persistent inflammation [14, 69]. In contrast to the ARDS_nonKTR group, the ARDS_KTR group exhibited a higher abundance of multiple immune cells, whereas the abundance of naïve T cells was lower. KTRs who suffer from impaired immune function resulting from the prolonged administration of immunosuppressive drugs [61] may develop T cell dysfunction [33, 40]. Viral infections have the potential to intensify this effect, ultimately resulting in the depletion of naïve T cells. It is noteworthy that the abundance of CD8^+^ T cells was higher in the ARDS_KTR group compared to that of the ARDS_nonKTR group. This implies that in KTRs, functionally mature T cells in the bloodstream may exhibit reduced tissue migration compared with those of non-KTRs with ARDS.

In B-cell immunity, extracellular mechanisms strive to compensate for the delayed or prolonged germinal center response by intensifying activities, leading to the highest measured levels of antibodies in the most severe COVID-19 cases [56]. Importantly, B_HLA-II, the dominant element in the B-plasma cell lineage of the control group, significantly decreased in both ARDS groups. This observation aligns with the previously described characteristics of SARS-CoV-2 infection, resulting in the dysregulation of intercellular crosstalk among adaptive immune cells [60]. When compared to the ARDS_nonKTR group, the diminished abundance of B_HLA-II in the ARDS_KTR group implied a greater degree of compromised B cell activation, indicating a potential link to the increased vulnerability of KTRs to COVID-19 [46]. We found that the ARDS_KTR group exhibit an enhanced plasma cell response compared with the ARDS_nonKTR group, leading to a greater production of antibodies for combating COVID-19. Proliferating and hyperactive plasma cells participate in the inflammatory responses and immune processes [7]. Nevertheless, this alteration may also result in the excessive release of inflammatory mediators, which, in turn, worsens tissue damage and exacerbates ARDS. Notably, unlike the ARDS_nonKTR group, the ARDS_KTR group exhibited a higher abundance of Plasma_IGHG3. In COVID-19 cases, somatic hypermutation (SHM) and class-switch recombination (CSR) events of IGHG3 are more frequent than those of other IgG subclasses [26]. High frequencies of SHM and CSR can specifically alter the hinge length polymorphism of IGHG3, diminishing the neutralizing ability of antibodies against the virus [34]. Hence, the elevated abundance of Plasma_IGHG3 in ARDS_KTR group could potentially exacerbate the risk of disease progression and mortality in these patients.

We observed that naïve T cells significantly decreased in both ARDS groups, which increases the likelihood of cytokine storms [47]. Terminally differentiated Naive.T_PCNA was found to be specifically enriched in both ARDS groups, likely resulting from DNA damage and genomic instability in host cells caused by viral infection [27, 36]. Previous studies indicate that the M protein of SARS-CoV-2 is capable of regulating the translocation of PCNA from the cell nucleus to the cytoplasm, and potentially sustains cell viability via PCNA ubiquitination during viral infection [52, 67]. Thus, viral infection can induce Naive.T_PCNA to engage in signaling pathways or ubiquitination, thereby contributing to DNA repair in host cells and facilitating viral replication. In the ARDS_KTR group, there was a significant increase in the abundance of PCNA-positive naïve T cells compared with the ARDS_nonKTR group. We postulate that the abundance of Naive.T_PCNA could indicate the proliferative potential of T cells in patients after viral infection under post-kidney transplantation immunosuppressive conditions [41, 50].

The marked reduction in CD8^+^ T cells in patients detrimentally affects the antiviral immune response [59], amplifying the severity of ARDS. It is noteworthy that both Te_GZMB_XAF1 and Te_CCL5_GZMH demonstrated elevated expression levels of GZMB and PRF1. GZMB and PRF1 act as crucial effector molecules in cytotoxic T lymphocytes, leading to acute and chronic rejection of solid organ transplants (Corrales-Tellez et al., 2013). It has been reported that PRF1 and GZMB expression in CD8^+^ T cells increases during COVID-19 progression, potentially causing host cell damage during viral clearance (Jiang et al., 2020). In this study, we observed that the T cells in the ARDS_KTR group were primarily composed of the Te_GZMB_XAF1 subpopulation. XAF1 enhances immune responses against RNA viruses by modulating chromatin accessibility [30]. Although Te_GZMB_XAF1 plays a vital role in antiviral responses, its elevated abundance may result in worsening the condition of KTRs. Furthermore, Te_GZMB_XAF1 had the potential to differentiate into Te_CCL5_GZMH, which formed a significant portion of CD8^+^ T cells in the ARDS_nonKTR group. Dysregulated CCL5 expression leads to chemotactic alterations and sustained inflammatory cascades mediated by cytokines, resulting in the development of ARDS [2]. Hence, Te_CCL5_GZMH could display a heightened effector phenotype, resulting in increased levels of chemokines and pro-inflammatory cytokines, potentially contributing to a more severe cytokine storm. In summary, we suggest that in KTRs with ARDS under immunosuppressive conditions and virus infection, impaired differentiation of Te_GZMB_XAF1 leads to its abnormal accumulation in the ARDS_KTR group. This could reduce the body's efficiency to clear the virus, hamper normal immune reconstitution, promote chronic inflammation formation, and ultimately worsen ARDS progression in KTRs.

Following SARS-CoV-2 infection, monocytes undergo metabolic reprogramming, manifesting bioenergetic changes and mitochondrial dysfunction [19]. We found that Mono_S100A12 constituted a major component in the monocytes of the ARDS_nonKTR group. Previous studies have demonstrated that S100A12 enhances the expression of adhesion factors and induces the activation of human monocytes via Toll-like receptor 4, thereby acting as an enhancer of innate immunity in the early stages of inflammation and sepsis [17, 29, 31, 37]. We observed that Mono_S100A12 was significantly enriched in cell adhesion molecules and the Toll-like receptor signaling pathway, suggesting its potential role in mediating immune dysregulation in the non-KTRs with ARDS through these pathways. Additionally, the interferon-inducible gene IFI27 is highly expressed in the respiratory tract and PBMCs of patients with COVID-19, and it may also affect the molecular mechanisms of the innate immune response through negative feedback regulation, thus facilitating viral replication [49, 58]. Our study revealed a significant enrichment of the Mono_IFI27 subpopulation in the ARDS_KTR cohort. The specific expression of IFI27 in this subpopulation may adversely modulate the immune response in KTRs, impairing viral clearance and the regulation of inflammatory processes. Consequently, Mono_IFI27 serves as an indicator of viral infection and immune activity in KTRs with ARDS and potentially plays a critical role in the pathogenesis of ARDS. Overall, the aberrant changes in monocyte subpopulations may further exacerbate the innate immune dysregulation in patients.

Previous studies have indicated that SARS-CoV-2 infection is not efficient in MDDCs [15]. The MDDC_AREG subpopulation was specifically enriched in both ARDS groups, and high expression of the pro-fibrotic gene AREG acts as a distinct marker for monocytes in severe COVID-19 cases [54, 55]. In diverse inflammatory and pathological contexts, AREG is expressed by a variety of activated immune cells, which coordinate mechanisms for tolerance and host resistance [51]. Our findings revealed enrichment of the MDDC_AREG subpopulation in the ErbB signaling pathway, which regulates cell survival, growth, and movement [66]. Consequently, MDDC_AREG may play a regulatory role in immune responses, tissue repair, and the modulation of inflammation in ARDS patients. Notably, MDDC_ANXA1 constituted a major component in the MDDC of the ARDS_KTR group. The expression of ANXA1 is markedly increased in the sera of critically ill COVID-19 patients [8]. Specific drugs, including dexamethasone, hydrocortisone, and prednisolone, have been used to manage severe cases of COVID-19 that target ANXA1 [32]. This indicates that the increased presence of MDDC_ANXA1 may contribute to mitigating the severity of COVID-19 and its related complications in immunosuppressed individuals.

In our study, we found that the receptor-ligand interaction pair CCL5-CCR1 was significantly activated in the ARDS_KTR group, potentially facilitating the migration and recruitment of immune cells. CCL5 has the ability to recruit lymphocytes to the sites of inflammation and allograft rejection, with CCR1 being one of its well-characterized ligands in various inflammatory diseases [10]. Previous studies have demonstrated the inhibitory effects of blocking CCL5-CCR1 on the development of acute and chronic cardiac allograft rejection [18, 65]. Thus, it is hypothesized that T cells expressing high levels of CCL5 facilitate monocyte infiltration in KTRs by interacting with the receptor CCR1 on the surface of monocytes, thereby promoting cellular rejection and contributing to the progression of ARDS.

Despite these findings, our study has some limitations. Our analysis is limited to PBMC samples from patients, and it is possible that distinct cellular changes occurred at the organ and tissue levels that were missed. Additionally, the clinical phenotypes of COVID-19-induced ARDS in patients who have undergone different types of organ transplantation can vary significantly. Finally, due to the unique nature of our sample types, we currently lack sufficient samples to conduct experimental validation at the mechanistic level.

## Conclusion

The key findings suggest that KTRs with ARDS exhibit a stronger delayed humoral response and elevated antibody levels attributed to abnormal changes in B cells. In the ARDS_KTR group, there may be an impaired differentiation function of Te_GZMB_XAF1, resulting in its abnormal enrichment in KTRs and thereby unfavorably affecting immune reconstitution in the body. Furthermore, the abnormal proliferation of monocytes leads to dysregulation of the innate immune system in KTRs with ARDS.

### Supplementary Information

**Additional file 1: Figure S1.** The intercellular communication of cytokines. cirPlot visualizing the intercellular communication events within cell subpopulations in patients with (A) COVID-19-induced ARDS and (B) COVID-19-induced ARDS after kidney transplantation. (C) CCL5 expression in naïve T cells and CD8^+^ T cells.

**Additional file 2: Table S1.** Clinical information of the samples.

## Data Availability

The datasets presented in this study can be found online at the National Genomics Data Center under the accession numbers HRA004752 and HRA005498.

## Abbreviations

ARDS: Acute respiratory distress syndrome
GRN: Gene regulatory network
KTRs: Kidney transplant recipients
MDDCs: Monocyte-derived dendritic cells
PBS: Phosphate-buffered saline
