# Supplementary figures and images for: Immune characteristics of kidney transplant recipients with acute respiratory distress syndrome induced by COVID-19 at single-cell resolution
Source: Respir Res. 2024 Jan 18;25:34. doi: 10.1186/s12931-024-02682-9 (PMC10795319; doi:10.1186/s12931-024-02682-9)

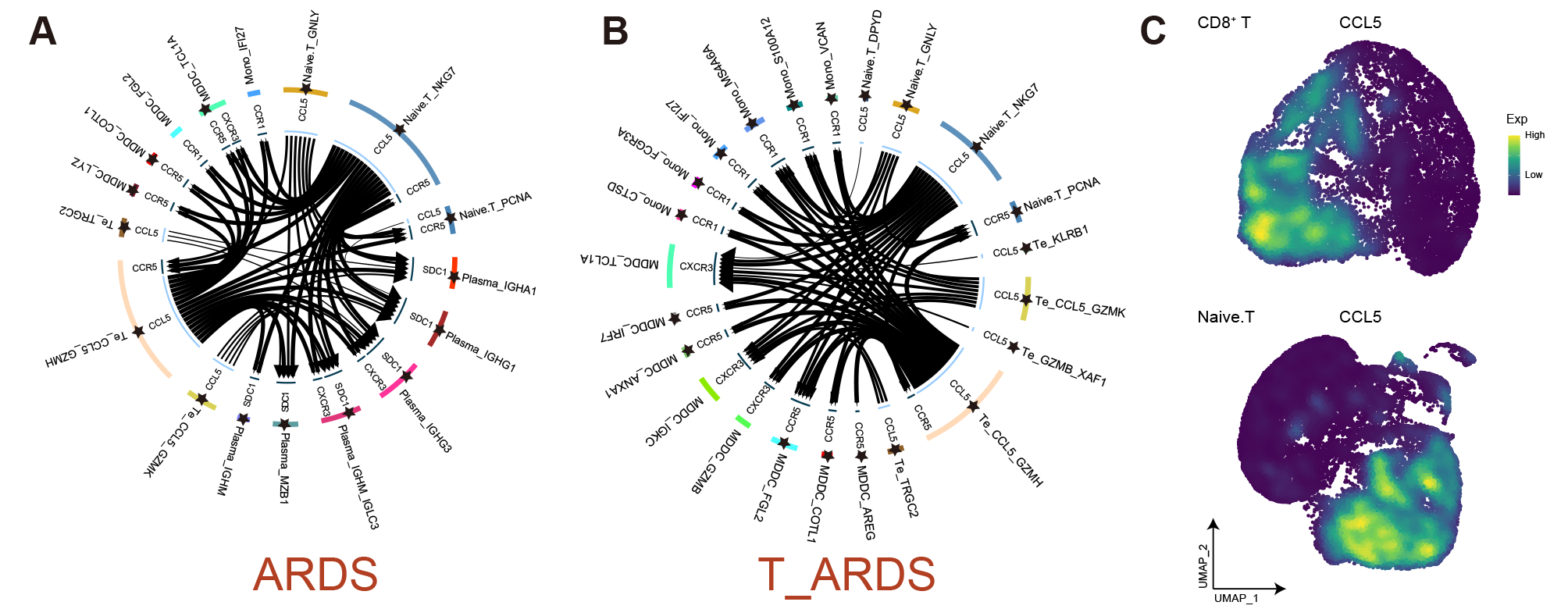

Supplement: Supplementary file 1 — Additional file 1: Figure S1. The intercellular communication of cytokines. cirPlot visualizing the intercellular communication events within cell subpopulations in patients with (A) COVID-19-induced ARDS and (B) COVID-19-induced ARDS after kidney transplantation. (C) CCL5 expression in naïve T cells and CD8+ T cells. [file 12931_2024_2682_MOESM1_ESM.tif]
